# The costs of overwintering in paper wasps (*Polistes dominula* and *Polistes gallicus*): the use of energy stores

**DOI:** 10.1007/s00360-024-01540-w

**Published:** 2024-03-05

**Authors:** Anton Stabentheiner, Teresa Mauerhofer, Regina Willfurth, Helmut Kovac, Edith Stabentheiner, Helmut Käfer, Iacopo Petrocelli

**Affiliations:** 1 Institute of Biology, University of Graz, Universitätsplatz 2, 8010 Graz, Austria; 2 Dipartimento di Biologia, Università di Firenze, Via Madonna del Piano, 6, 50019 Sesto Fiorentino, Italy

**Keywords:** Paper wasps, Polistes, Overwintering costs, Energy stores, Lipids, Carbohydrates

## Abstract

Overwintering insects are facing energetic challenges because of food shortage, low temperature, and desiccation stress. Paper wasps of the genus *Polistes* overwinter as mated adults (gynes) in hibernacula protecting them from predation, snow, and rain but barely from low environmental temperature. In different climates, they face differing overwintering temperature regimes, and therefore they may differ in their energy use. We investigated how much of energy resources built up until autumn is used during diapause dormancy in natural hibernacula by measuring lipid, glycogen, and free carbohydrate content in autumn and early spring in *Polistes dominula* from temperate European (Austrian) and warm Mediterranean (Italian) climate and *Polistes gallicus* from Mediterranean climate. Winter energy consumption amounted to ~ 339 and ~ 310 J per wasp in the Austrian and Italian *Polistes dominula* populations. The smaller Italian *Polistes gallicus* consumed ~ 247 J. This amounts to 2.62, 2.35, and 1.79 J per day. Of this, the energy demand was mainly fuelled by lipids (84%, 93%, and 90%, respectively), but glycogen stores contributed also considerably (16%, 6%, and 9%). Free carbohydrates decreased only by 0.7%, 1%, and 0.8%. While fat stores seem still sufficient in spring, the wasps depleted most of their carbohydrates. The energy reserves of 396, 400, and 147 J per wasp remaining in spring in the three populations seem sufficient to fuel rest or simple brood care activities for a whole summer but restrict foraging flights to a few hours (~ 3.5–6 h). Results suggest that energy supply might become challenging in expected future climate scenarios.

**Supplementary Information:**

The online version contains supplementary material available at 10.1007/s00360-024-01540-w.

## Introduction

*Polistes dominula* and *Polistes gallicus* are two closely related paper wasp species, which differ in their ranges of distribution but in part settle in overlapping areas (Neumeyer et al. 2014). Both species are suggested to originate from the Mediterranean climate region (Schmid-Egger et al. 2017). However, while *P. gallicus* remained in the Mediterranean climate, *P. dominula* expanded its range of settlement to temperate climates (Neumeyer et al. 2014; Schmid-Egger et al. 2017; Kovac et al. 2017). Meanwhile, *P. dominula* was also introduced to North America where they inhabit similar habitats than in Eurasia (Cervo et al. 2000). In Polistine wasps, it is the mated gynes (imagines) which overwinter in sheltered places called hibernacula (see e.g., Dapporto et al. 2004; Kovac et al. 2022b). In late autumn, they enter a dormant stage called diapause, where they do not feed and remain quite calm. In typical winter hibernacula, the temperature decreases until midwinter, and then rises again (Kovac et al. 2022b, 2023). In spring, the gynes found a new colony and are then called queens.

Overwintering insects are facing energetic challenges because of e.g., missing access to food, low temperature and desiccation stress (e.g., Denlinger and Lee 2010; Lee and Denlinger 1991; Overgaard et al. 2007; Sinclair 2015; Enriques and Visser 2023; Turnbull et al. 2023). Finding proper places for overwintering, adapting physiologically to winter environmental conditions, and keeping a diapause help to reduce the consumption of energy reserves. In diapause, the reduction of behavioral and metabolic activity and reproduction arrest, and the approximately exponential decrease of metabolism with temperature helps insects to save energy on the one hand. On the other hand, due to the often long duration of diapause, depletion of energy stores may be critical, because the metabolic resources built up in autumn are limited. The prerequisite for successful nest founding and breeding in spring is the accumulation of sufficient energy reserves in autumn. However, different climates may have differing effects on the energy reserves available in spring. Hahn and Denlinger (2007) pointed out that in insects, “the energy reserves expended during diapause have a profound effect on post-diapause fitness”. In paper wasps, these energy reserves have to allow for building up and maintaining protection against the cold, for use in winter metabolism, and for founding a new colony in spring (compare Sinclair 2015).

The costs of overwintering can be estimated in different ways. One possibility is by calculation from fit curves relating respiration to ambient or body temperature, and recordings of temperature in the winter hibernacula or of environmental temperature (Williams et al. 2012; Roberts and Williams 2022; Kovac et al. 2022b, 2023). Another possibility is to directly measure the depletion of energy stores (e.g., Lehmann et al. 2016; Shi et al. 2023). Investigations on winter depletion of insect energy stores mostly refer to depletion of fat reserves (Fliszkiewicz et al. 2012; Vesterlund et al. 2014). Many tissues, however, rely also on sufficient supply with carbohydrates, the use of which is often unknown. Though the main organ of energy store is the fat body (e.g., Lee and Denlinger 1991; Sinclair 2015; Shi et al. 2023), the reserves in other tissues and in body fluids may be important to survive a long winter and allow for proper nest building in spring. Therefore, we here determined the depletion of total fat and glycogen reserves, and the amount of soluble carbohydrates. The direct measurement of energy resources is used to estimate how long the wasps are able to survive with the reserves remaining after a long winter, and the time to maintain foraging activity.

Polistine wasps from different climates, however, face differing winter temperatures, and this way may differ in their winter energy use (Kovac et al. 2022b, 2023). Since the costs of overwintering may also differ between species because of specific adaptation to their local climate (Lee and Denlinger 1991; Sinclair 2015; Kovac et al. 2020, 2023), we hypothesize that species and populations may differ in their winter energy use. Therefore, we here compare the costs of overwintering and spring energy reserves between *Polistes dominula* inhabiting temperate Central European (Austrian, AT) and warm Mediterranean (Italian, IT) climate, and *Polistes gallicus* inhabiting Mediterranean (IT) climate only.

## Materials and methods

### Animals

The energy reserves of gynes of three paper wasp populations from Austria (AT) and Italy (IT) were determined before and after overwintering *(Polistes dominula* AT, *Polistes dominula* IT, *Polistes gallicus* IT). Wasp gynes were collected from their winter hibernacula in the field, in Gschwendt and Fernitz (near Graz, Austria; *P. dominula* AT) and Pistoia and Trespiano (near Firenze, Italy; *P. dominula* IT, *P. gallicus* IT) in autumn 2018 and 2019 and spring 2019, 2020, and 2021 (see Table S3). The wasps originated from at least 5 different natural hibernacula per population (Figs. S1, S2). After weighing, they were stored in Eppendorf vials at – 80 °C.

### Determination of lipids, glycogen, and free (soluble) carbohydrates

The determination of energy resources (lipids, glycogen, and free carbohydrates) followed the protocol of Lee (2019), with some adaptations according to Lorenz (2003) and Guckert and White (1988). A detailed, step-by-step description can be found in the Supplementary materials.

#### Sample preparation

The frozen wasps were equilibrated to room temperature for 1 h in the Eppendorf vials. Afterward their fresh mass (FM) was determined. After drying at 55 °C for 3 days in an oven with recirculating air, their dry mass (DM) was measured.

Before further analysis, the wasps were washed in *n*-hexane for 30 s and dried for 20 min, to wash off cuticular lipids (to improve determination of storage lipids). Every wasp was cut to small pieces with scissors in the Eppendorf vial, and then milled in the vial with three steel bullets (diameter 2 mm) in a Retsch mill for 2 min at 30 Hz (room temperature), resulting in a fine-grained powder promising optimal extraction.

#### Extraction

For lipid and free carbohydrate extraction, 600 µl of extraction reagent and 100 µl Na_2_SO_4_ were added to each vial, which afterward was vortexed for 30 s, shaken for 5 min in a thermomixer, and then centrifuged for 15 min at 14,000 rpm (room temperature). The supernatant was pipetted in a new 2 ml Eppendorf vial. The pellet was extracted again with 400 µl reagent, the supernatant added to the first supernatant and filled up to 1 ml if necessary, and stored at 4 °C. The combined supernatant was divided in two parts (1:1 for lipids and free carbohydrates), and dried by evaporation in a Speed Vac (temperature set to “low”) for 4 h.

The pellet (solid phase) in the original extraction vial was dried at 50 °C in a thermomixer, and used for glycogen determination. All Eppendorf vials were purged with nitrogen (N_2_) and stored at –80 °C for later use.

#### Lipids

Lipid content was determined with the sulfophosphovanillin (PV) reaction according to Park et al. (2016). Its high reliability was proved by Williams et al. (2011). As a calibration standard, we used a mix of 1 mg ml^−1^ tripalmitin (C_51_H_98_O_6_) 41%, 1 mg ml^−1^ triolein (C_57_H_104_O_6_) 36%, and 1 mg ml^−1^ trilinolenin 23% (C_57_H_98_O_6_) in *n*-hexane (C_6_H_14_) (Williams et al. 2011). For lipid extraction, we used a mixture of hexane and isopropanol (3:2) according to Guckert et al. (1988), Guckert and White (1988) and Palmquist and Jenkins (2003), instead of chloroform and methanol (Lee 2019; Lorenz 2003). Hexane and isopropanol extract a smaller portion of polar lipids, and this way of phospholipids in cell membranes, in comparison to chloroform and methanol (Guckert et al. 1988; Guckert and White 1988; Palmquist and Jenkins 2003), and this way improve determination of storage lipids. The fine-grained reaction powder produced with the Retsch mill promised an accurate extraction of storage lipids.

The wasp samples and a lipid standard in concentrations of 0, 10, 20, 30, and 40 µg ml^−1^ were measured in disposable PMMA cuvettes in a photometer at 530 nm wavelength, with air as a reference.

#### Glycogen

Glycogen content was determined with the Anthrone reaction (e.g., Lee 2019; and literature quoted there). The pellet (solid phase) of the original extraction was washed with 400 µl methanol, vortexed for 30 s, and then centrifuged for 5 min at 10,000 rpm. The supernatant was discarded and the procedure was repeated.

For calibration, a glycogen standard (100 µl) was prepared freshly, and pipetted into 2 ml Eppendorf vials in concentrations of 0, 25, 50, and 100 µg ml^−1^. 100 µl of the samples as well as of the different concentrations of the calibration standard was pipetted into graded glass vials and filled up to 5 ml with the Anthrone reagent. After incubating the glass vials for 15 min at 90 °C, they were cooled down to room temperature (~ 23 °C) with cold water. The solution was transferred to disposable PMMA cuvettes and measured in a photometer at 620 nm, with air as a reference.

#### Free carbohydrates

Free (soluble) carbohydrates were determined with the Anthrone reaction (e.g., Lee 2019). 500 µl of Aqua bidest was added to the second part of the combined supernatant from the sample preparation (the other half was used for lipid determination), vortexed for 1 min, and the sample allowed to dissolve for 5 min in an ultrasonic bath. 100 µl of the resulting milky turbid solution was pipetted into 2 ml reaction vials and added by 1900 µl of Anthrone reagent.

The glucose standard was pipetted into 2 ml Eppendorf vials in concentrations of 0, 10, 20, 30, and 40 µg ml^−1^, and filled up to 2 ml with Anthrone reagent for generating a calibration curve in the photometer. The sample and the standard solutions were measured in disposable PMMA cuvettes in a photometer at 620 nm, with air as a reference.

### Respiratory quotient (RQ)

A mean winter respiratory quotient (RQ_Winter_) was estimated as a weighted mean of measured lipid (RQ = 0.7), glycogen, and free carbohydrate (RQ = 1) changes between seasons (compare Erregger et al. 2017), by multiplying the RQ values by the mass of the respective energy stores, divided by the sum of masses. In addition, we included structure mass changes as a rough estimate of protein degradation (RQ = 0.8).

In order to allow a proper estimation of the time the wasps are able to survive with the energy reserves remaining in spring, we determined the respiratory quotient in summer individuals (workers) (RQ_Summer_) of *P. dominula* AT and *P. gallicus* IT and, for comparison, in *P. biglumis* AT, according to the measurements in overwintering gynes by Kovac et al. (2022b). In short, seven wasps were placed individually in 2.23 ml respiration measurement chambers for about 2 h (to accumulate enough CO_2_ and O_2_-depleted air) and measured at 25 °C ambient temperature. Care was taken to have long enough measurement periods to include several respiratory cycles of resting individuals (see Käfer et al. 2015), which avoids unrealistically low RQ values during discontinuous respiration. An additional empty chamber served as a reference for control of instrument drift. A RM gas flow multiplexer (Sable Systems International, Las Vegas, USA) passed commercial dried air to a reference and a measurement channel (parallel mode) of an Uras 14 differential infrared gas analyzer (DIRGA; ABB, Zürich, Switzerland) followed by an Oxzilla 2 differential oxygen analyzer (Sable Systems). The air flow was regulated at 144 ml min^−1^ by factory-calibrated Brooks 5850 S mass flow controllers. The multiplexer flushed the eight measurement chamber channels in sequential order. The air leaving the measurement chambers was dried with Drierite^®^ desiccant (Hammond Drierite Co. Ltd., Xenia, USA) before it entered the DIRGA and the Oxzilla. The Uras 14 CO_2_ analyzer was calibrated against internal calibration cuvettes, and the Oxzilla 2 O_2_ analyzer against air from outside the laboratory, before and after measurement (Stabentheiner et al. 2012). Any instrument drift and offset was compensated during evaluation according to the difference between the measurement channels and the reference channel. Data acquisition and evaluation was done with the DIRGA CO_2_ gas analyzer system software (Centrol 5; Harnisch, Austria). The readout of dried air was integrated against time. In this way, the respiratory quotient was calculated as RQ = ∫CO_2_/∫O_2_.

### Statistics

Statistics was done with IBM SPSS (IBM Corporation) and Statgraphics Centurion 18 (Statgraphics Technologies, Inc.). ANOVA was applied to figure out the effects of population and season on the amount and use of energy reserves. The Mann–Whitney *U* test was used for comparisons of resource (lipids, glycogen, and free carbohydrates) and total energy content between populations and seasons. ANOVA contrasts were used to test for differences in the winter energy use between the sample populations.

## Results

A main goal of the present study was to determine the wasps’ energy reserves before and after natural overwintering. For this purpose, we let the wasps overwinter in their natural, self-selected hibernacula without disturbing them. This way they experienced the natural course of diurnal and seasonal temperature changes, and impairment of physiology and behavior was prohibited (compare Jandt et al. 2015). In order to keep the local populations alive and vital, care had to be taken with the amount of wasps collected from the limited number of hibernacula at a certain location. Therefore, sample sizes differed between locations and seasons.

### Mass and water content

**Fig. 1:**
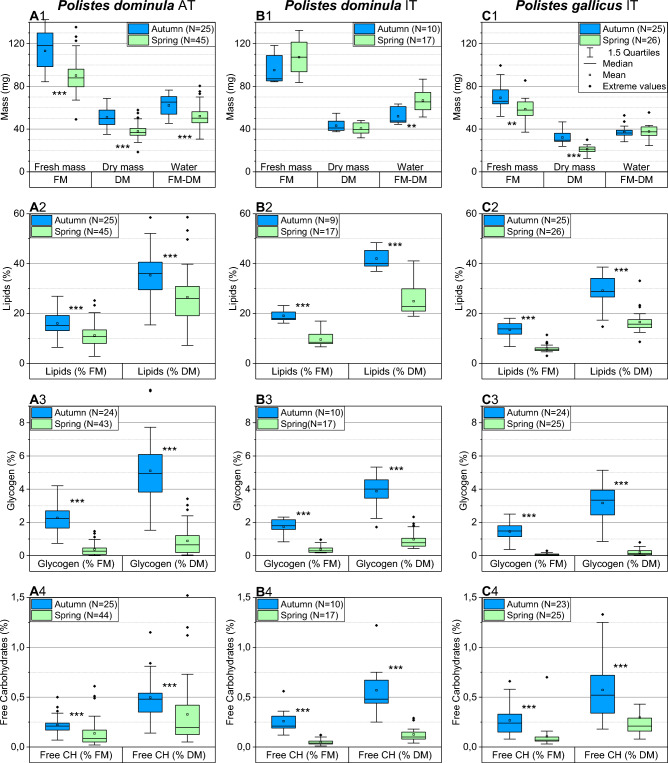
Change of mass (weight) and water content, and of total body sources of energy (content of lipids, glycogen and free (soluble) carbohydrates), between autumn and spring of three *Polistes* populations from temperate Austrian (AT) and Mediterranean Italian (IT) climate. Sources of energy calculated as percentage of fresh mass (FM) or dry mass (DM). FM-DM: water content. Boxes show medians with 1st and 3rd quartile, mean (square), ± 1.5 quartiles (whiskers), and extreme values (> ± 1.5 quartiles, small rhombi). **P < 0.01, ***P < 0.001; Mann–Whitney *U* test). Compare Table 1, and Supplementary Tables S1, S4, and S5

The mass of the wasps differed between the populations and species. In autumn, the mean fresh mass (FM) of *Polistes dominula* AT, *P. dominula* IT, and *P. gallicus* IT amounted to 113.1, 95.2, and 69.3 mg, respectively. This changed to 90.0, 107.3, and 58.7 mg in spring (Fig. 1-A1,-B1,-C1; Table 1). The mean dry mass (DM) of *P. dominula* AT, *P. dominula* IT, and *P. gallicus* IT changed from 51.0, 43.2, and 32.1 mg in the autumn samples to 37.9, 40.5, and 21.1 mg in spring. The mean water content (FM-DM) changed from 62.1, 52.0, and 37.2 mg in autumn to 52.1, 66.8, and 37.7 mg in spring, respectively (Fig. 1).

**Table 1:** Descriptive statistics of energy resource determination in paper wasps *Polistes dominula* and *Polistes gallicus* from Austria (AT) and Italy (IT) (N = 148 wasps)

Species/population	Season	Content per wasp (mg)	Min	*Q*1	Median	*Q*3	Max	Mean	SD	*N*
*P. dominula* AT	Autumn	Fresh mass (FM)	84.3	96.7	118.1	129.9	142.5	**113.1**	17.6	25
		Dry mass (DM)	35.1	43.4	50.0	59.0	68.7	**51.0**	9.1	25
		Water content (FM-DM)	45.4	53.8	65.3	70.4	76.5	**62.1**	9.7	25
		Structure mass (SM)	14.30	25.75	27.70	35.70	48.40	**30.35**	8.13	25
		Lipids	8.20	12.70	17.70	22.10	33.30	**17.86**	5.90	25
		Glycogen	0.93	1.65	2.24	3.38	5.08	**2.63**	1.31	24
		Free carbohydrates	0.07	0.17	0.22	0.30	0.60	**0.25**	0.13	25
	Spring	Fresh mass (FM)	49.1	78.9	87.9	98.0	135.4	**90.0**	17.3	45
		Dry mass (DM)	18.5	34.1	36.8	40.7	57.8	**37.9**	7.2	45
		Water content (FM-DM)	30.6	45.7	50.2	57.8	80.4	**52.1**	10.9	45
		Structure mass (SM)	14.30	23.75	27.10	30.95	46.30	**27.35**	6.43	45
		Lipids	2.70	6.70	9.40	13.25	21.80	**10.05**	4.28	45
		Glycogen	0.01	0.06	0.23	0.49	1.73	**0.37**	0.42	43
		Free carbohydrates	0.02	0.04	0.07	0.17	0.61	**0.13**	0.13	44
*P. dominula* IT	Autumn	Fresh mass (FM)	84.3	85.1	87.1	109.8	118.3	**95.2**	13.2	10
		Dry mass (DM)	37.5	38.6	41.0	48.4	54.8	**43.2**	6.0	10
		Water content (FM-DM)	44.4	46.0	47.6	61.1	63.5	**52.0**	7.6	10
		Structure mass (SM)^a^	19.20	19.83	21.75	27.68	52.50	**25.49**	10.09	10
		Lipids	15.00	16.25	17.50	19.25	20.00	**17.51**	1.70	9
		Glycogen	0.71	1.35	1.74	2.13	2.53	**1.68**	0.56	10
		Free carbohydrates	0.10	0.17	0.21	0.28	0.63	**0.25**	0.15	10
	Spring	Fresh mass (FM)	83.6	93.5	107.3	122.7	132.3	**107.3**	15.6	17
		Dry mass (DM)	31.8	35.7	39.7	45.8	48.0	**40.5**	5.4	17
		Water content (FM-DM)	51.3	56.5	65.4	76.3	86.7	**66.8**	11.2	17
		Structure mass (SM)^a^	21.70	25.90	29.60	35.20	36.80	**29.97**	4.82	17
		Lipids	7.10	7.60	8.90	12.25	15.80	**10.10**	2.69	17
		Glycogen	0.20	0.20	0.30	0.50	0.90	**0.40**	0.26	17
		Free carbohydrates	0.02	0.03	0.04	0.07	0.11	**0.05**	0.03	17
*P. gallicus* IT	Autumn	Fresh mass (FM)	51.9	63.2	66.0	77.1	99.5	**69.3**	11.3	25
		Dry mass (DM)	23.8	28.3	29.6	36.7	46.7	**32.1**	6.3	25
		Water content (FM-DM)	28.1	34.3	36.3	39.8	52.8	**37.2**	5.2	25
		Structure mass (SM)	14.90	18.35	20.40	24.65	28.50	**21.48**	3.93	25
		Lipids	4.30	7.25	8.80	10.75	18.00	**9.44**	3.16	25
		Glycogen	0.24	0.75	0.94	1.30	1.93	**1.04**	0.46	24
		Free carbohydrates	0.05	0.10	0.17	0.28	0.51	**0.20**	0.13	23
	Spring	Fresh mass (FM)	37.1	52.7	57.8	65.4	85.5	**58.7**	9.3	26
		Dry mass (DM)	12.6	18.8	21.2	23.3	30.0	**21.1**	3.5	26
		Water content (FM-DM)	24.5	34.1	37.5	42.1	55.5	**37.7**	6.1	26
		Structure mass (SM)	10.60	15.63	18.00	19.45	25.20	**17.47**	3.00	26
		Lipids	1.80	2.88	3.15	4.33	6.40	**3.49**	1.08	26
		Glycogen	0.00	0.00	0.00	0.10	0.20	**0.03**	0.06	21
		Free carbohydrates	0.02	0.03	0.04	0.07	0.48	**0.07**	0.09	25

*Q*1, *Q*3 = quartiles 1 and 3; Min, Max = minimum and maximum values; means in bold printing

^a^Structure mass (SM) change between seasons was assumed as ~ 3 mg in *P. dominula* IT for calculation of daily energy use (Fig. 3, Table 3), because of a 1-year delay of spring sampling due to legal restriction (lockdown) in Italy 2020. For percent values, see Supplementary Table S1

In addition, we calculated the ‘structure mass’ (SM), which is the dry mass minus the mass of the summed main energy stores (lipids, glycogen, and free carbohydrates). It changed from 30.35, 25.5, and 21.5 mg in autumn to 27.35, 30.0, and 17.5 mg in spring, in *P. dominula* AT, *P. dominula* IT, and *P. gallicus* IT, respectively (Tables 1, S1).

### Seasonal change of energy stores

#### Lipids

The main energy stores were the lipids. In *P. dominula* AT, *P. dominula* IT, and *P. gallicus* IT, their mean contents per wasp changed from 17.86, 17.51, and 9.44 mg in the autumn samples to 10.05, 10.1, and 3.49 mg in spring, respectively (Tables 1, 2). This amounted to 16.01%, 19.07%, and 13.47% of fresh mass in autumn, and 11.21%, 9.56%, and 5.95% in spring (Fig. 1-A2,-B2,-C2). In relation to dry mass, this was 35.32%, 42.02%, and 29.13% in autumn, and 26.51%, 24.95%, and 16.58% in spring in the three populations, respectively (Table S1).

**Table 2:** Resources of total lipids, glycogen, and free carbohydrates, and their use during overwintering, in *Polistes* populations from Austria (AT) and Italy (IT)

Species/population	Mean content (mg)		Winter resource use		Winter energy use
	Autumn	Spring	(mg)	(%) of autumn	(%) of total J per population
	Lipids				
*Polistes dominula* AT	17.86	10.05	7.81	43.72	83.57
*Polistes dominula* IT	17.51	10.10	7.41	42.32	92.66
*Polistes gallicus* IT	9.44	3.49	5.95	63.01	90.14
	Glycogen				
*Polistes dominula* AT	2.63	0.37	2.26	85.77	15.74
*Polistes dominula* IT	1.68	0.40	1.28	76.19	6.33
*Polistes gallicus* IT	1.04	0.03	1.01	97.12	9.10
	Free carbohydrates				
*Polistes dominula* AT	0.25	0.13	0.13	49.33	0.69
*Polistes dominula* IT	0.25	0.05	0.20	80.00	1.01
*Polistes gallicus* IT	0.20	0.07	0.13	66.11	0.76

For detailed descriptive statistics see Table 1, and for the effects of population and season on resource contents see Table S2

#### Glycogen

Mass of glycogen reserves amounted to only about 9.6–15% of lipid stores in autumn and to 0.9–4% in spring. In *P. dominula* AT, *P. dominula* IT, and *P. gallicus* IT, their mean contents per wasp changed from 2.63, 1.68, and 1.04 mg in autumn to 0.37, 0.40, and 0.03 mg in spring, respectively (Tables 1, 2). This amounted to 2.28%, 1.75%, and 1.46% of fresh mass in autumn, and 0.37%, 0.38%, and 0.07% in spring (Fig. 1-A3,-B3,-C3). In relation to dry mass, this was 5.11%, 3.88%, and 3.14% in autumn, and 0.89%, 0.99%, and 0.20% in spring in the three populations, respectively (Table S1).

#### Free (soluble) carbohydrates

The smallest amount of the main energy reserves was the free (soluble) carbohydrates. They did not change as much as lipids and glycogen between seasons. Their mass amounted to only 1.4–2.1% of lipid stores in autumn, and to 0.5–2% of lipid stores in spring. In *P. dominula* AT, *P. dominula* IT, and *P. gallicus* IT, their mean contents per wasp changed from 0.25, 0.25, and 0.20 mg in the autumn samples to 0.13, 0.05, and 0.07 mg in spring, respectively (Table 1). This amounted to 0.22%, 0.26%, and 0.27% of fresh mass in autumn, and 0.14%, 0.05%, and 0.11% in spring (Fig. 1-A4,-B4,-C4). In relation to dry mass, this was 0.50%, 0.57%, and 0.57% in autumn, and 0.33%, 0.13%, and 0.30% in spring in the three populations, respectively (Table S1).

### Winter energy use

**Fig. 2:**
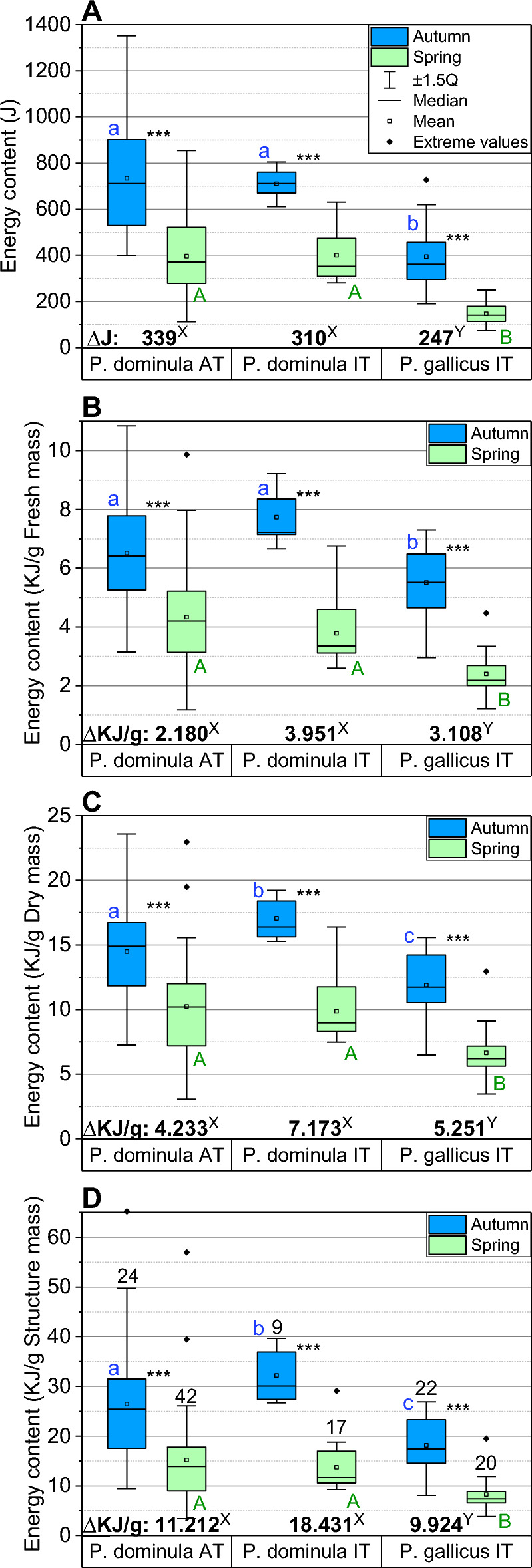
Comparison of the total energy content (lipids, glycogen and free carbohydrates) between *Polistes dominula* AT, *P. dominula* IT, and *P. gallicus* IT, and mean energy consumption (Δ*J*) during winter (see also Tables 2 and 3). **A**–**D**: Boxes show medians with 1st and 3rd quartile, mean (square), ± 1.5 quartiles (whiskers), and extreme values (> ± 1.5 quartiles, small rhombi). Different letters at the boxes show significant differences between populations in autumn (small, blue; *P* < 0.001, except a:b and a:c in parts **C** and **D**, *P* < 0.05) and spring (capital letters, green; all *P* < 0.001), and of energy consumption between species (Δ*J*; pairwise ANOVA, *P* < 0.05; see Table 4); ***P < 0.001 between seasons (Mann–Whitney *U* test)

Different fuels contribute differently to the energy content. We used a calorific value of 38.9 kJ g^−1^ for lipids and 15.7 kJ g^−1^ for carbohydrates (glucose). The main source of energy was lipids, accounting for 83.6% to 92.7% of the total energy consumption, whereas glycogen amounted to only 9.1–15.7% (Table 2). Soluble carbohydrates changed by only 0.69–1.01% of the total energy consumption during the winter. Mean total energy consumption, estimated from changes in fat and carbohydrate content, amounted to 339 J in Austrian *P. dominula* AT and to 310 J in Italian *P. dominula* IT. The smaller Italian *P. gallicus* IT consumed only 247 J on average (Fig. 2, Table 3). This equals a consumption of 46.12%, 43.69%, and 62.68% of autumn reserves, respectively (Table 3). The energy reserves remaining in spring amounted to 396 J in *P. dominula* AT, 400 J in *P. dominula* IT and 147 J in *P. gallicus* IT, which is 53.9%, 56.3%, and 37.3% of autumn reserves (Fig. 2, Table 3).

**Table 3:** Energy reserves calculated from lipids and carbohydrates in autumn and spring, and energy consumption in relation to fresh mass (FM), dry mass (DM), and structure mass (SM = dry mass minus mass of lipids and carbohydrates), of *Polistes* populations overwintering in Austrian (AT) or Italian (IT) climate

Species/population	Mean energy reserves			Mean energy consumption					Daily energy consumption	Duration of season
	Autumn	Spring	Spring							
	(J) ± SD/*N*	(J) ± SD/*N*	(%) of autumn	(J)	(%) of autumn	(J/g) of FM	(J/g) of DM	(J/g) of SM	(J/day)	(days)
*P. dominula* AT	734.30 ± 234.99/24	395.62 ± 165.89/42	53.88	338.68	46.12	2,180.0	4,233.1	11,212.1	2.62	129.5
*P. dominula* IT	710.48 ± 67.43/9	400.08 ± 107.45/17	56.31	310.40	43.69	3,950.9	7,173.1	18,431.1	2.35	132
*P. gallicus* IT	393.97 ± 131.17/22	147.04 ± 42.58/20	37.32	246.93	62.68	3,108.4	5,251.2	9,924.3	1.79	138

Only values of wasps averaged, where all three energy resources could be measured in the same individual (lipids, glycogen, and free carbohydrates). For statistics on energy consumption see Table 4. For sampling dates (duration of seasons) see Table S3

ANOVA showed that both season (i.e., autumn and spring) and population had a highly significant effect on mass-specific energy content of lipid and glycogen stores (*P* << 0.0001; Table S2). However, the effect of both factors could not be clearly separated in most cases (i.e., there were interactions). Concerning the content of free (soluble) carbohydrates only season had an effect but not population, with no interaction between both factors (Table S2; see also Table S5).

We could observe interspecific and intraspecific differences of the *total* energy costs of hibernation. ANOVA revealed significant effects of both population and season on mass-specific energy content (*P* << 0.0001; Table 4). There were no interactions between season and population.

**Table 4:** ANOVA of total energy content (lipids and carbohydrates), in J/wasp, and J/g of fresh mass (FM), dry mass (DM), and structure mass (SM)

	Energy content	Square sums	*df*	Mean squares	*F*-quotient	Difference	+/− Limits	*P* <
	**(J) per wasp**							
Main effects	Population	2.35491E6	2	1.17746E6	49.89			0.0000
	Season	2.90484E6	1	2.90484E6	123.09			0.0000
Contrasts	*P. dominula AT—P. dominula* IT					5.95628	70.3768	n.s.
	*P. dominula AT—P. gallicus* IT					290.934	60.61	0.05
	*P. dominula IT—P. gallicus* IT					284.978	76.4452	0.05
	**(J/g) of fresh mass (FM)**							
Main effects	Population	6.93169E7	2	3.46584E7	16.13			0.0000
	Season	2.51012E8	1	2.51012E8	116.85			0.0000
Contrasts	*P. dominula AT—P. dominula* IT					− 77.7371	671.463	n.s.
	*P. dominula AT—P. gallicus* IT					1546.46	578.278	0.05
	*P. dominula IT—P. gallicus* IT					1624.2	729.361	0.05
	**(J/g) of dry mass (DM)**							
Main effects	Population	3.33258E8	2	1.66629E8	16.71			0.0000
	Season	8.2834E8	1	8.2834E8	83.08			0.0000
Contrasts	*P. dominula AT—P. dominula* IT					− 657.039	1446.58	n.s.
	*P. dominula AT—P. gallicus* IT					3215.15	1245.82	0.05
	*P. dominula IT—P. gallicus* IT					3872.19	1571.31	0.05
	**(J/g) of structure mass (SM)**							
Main effects	Population	1.87078E9	2	9.35392E8	13.59			0.0000
	Season	4.65332E9	1	4.65332E9	67.61			0.0000
Contrasts	*P. dominula AT—P. dominula* IT					− 1049.49	3800.57	n.s.
	*P. dominula AT—P. gallicus* IT					7814.39	3273.13	0.05
	*P. dominula IT—P. gallicus* IT					8863.88	4128.28	0.05

Population (corrected for the effect of season): *Polistes dominula* AT, *Polistes dominula* IT, and *Polistes gallicus* IT. Season (corrected for the effect of population): autumn and spring. There were no interactions between main effects (population and season). Contrasts between populations calculated with Fisher’s LSD method. Only measurements included where all three energy resources (lipids, glycogen, free carbohydrates) could be measured in the same wasp (*N* = 134 wasps)

Mean total energy costs of overwintering were nearly identical in *P. dominula* overwintering in the Austrian (1.09 times higher) and in the Italian climate, and 1.37 times higher in the Austrian *P. dominula* in comparison to the Italian *P. gallicus* (compare Table 3). Pairwise ANOVA comparisons supported this finding (see contrasts in Table 4). If one takes a glance at the mass-specific overwintering costs, however, the relation of means reverses (compare Fig. 2 and Table 3). Fresh (wet) mass-specific costs were 1.81 times higher in *P. dominula* overwintering in the Italian than in the Austrian climate, and 1.42 times higher in the Italian *P. gallicus* in comparison to the Austrian *P. dominula*. Dry mass-specific energy costs of overwintering were 1.69 times higher in *P. dominula* overwintering in the Italian than in the Austrian climate, and 1.24 times higher in the Italian *P. gallicus* in comparison to the Austrian *P. dominula*. However, pairwise ANOVA contrasts revealed that mass-specific differences were only significant between *P. gallicus* IT and both *P. dominula* AT and *P. dominula* IT but not between *P. dominula* AT and *P. dominula* IT (Table 4).

**Fig. 3:**
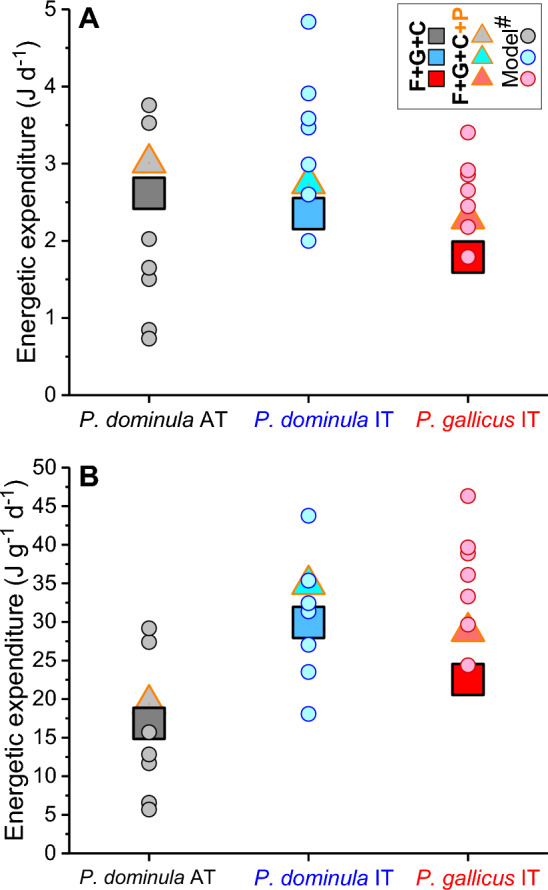
Mean daily energetic expenditure during the winter season of *Polistes dominula* AT, *P. dominula* IT, and *P. gallicus* IT. **A** In Joule per day, and **B** in Joule per day and gram fresh mass (FM). Mean duration of season = 133.2 days (Table 3, Table S3). F + G + C = lipids, glycogen, and free carbohydrates (squares); F + G + C + P = lipids + glycogen + free carbohydrates + estimated protein degradation (triangles); in comparison to ^#^model calculations by Kovac et al. (2023) from measurements of respiration and temperature recordings in winter hibernacula (circles)

From the contents of lipids and total carbohydrates in autumn and spring (Table 2), and the difference between sampling dates (Table S3; duration of season in Table 3), we calculated a mean daily energy expenditure of 2.62, 2.35, and 1.79 J per day in *P. dominula* AT, *P. dominula* IT and *P. gallicus* IT, respectively (Table 3, Fig. 3).

### Respiratory quotient (RQ)

A mean winter respiratory quotient (RQ_Winter_), estimated as a weighted mean of lipid, glycogen and free carbohydrate mass changes between seasons (Table 1), amounted to RQ_Winter_ = 0.77, 0.75 and 0.75 for *Polistes dominula* AT, *P. dominula* IT and *P. gallicus* IT, respectively.

The respiratory quotient of summer individuals (RQ_Summer_) was determined as 1.04 (SD = 0.07, *N* = 7) in *P. dominula* AT and as 0.99 in *P. gallicus* IT (SD = 0.09, *N* = 7). For comparison, in the mountainous species *P. biglumis* AT, we measured a quite similar value: RQ = 1.05 (SD = 0.06, *N* = 7). This suggests that summer individuals of different species generally consume mainly carbohydrates as energetic fuels.

## Discussion

Paper wasp gynes mate in autumn and overwinter as adults, similar to vespine wasps and bumblebees. The overwintering as adults makes them ready for immediate nest building and brood care in spring. Winter energy use can be assessed indirectly by calculation from known relationships of respiratory metabolism on ambient temperature combined with measurements of hibernaculum temperature during the winter (Kovac et al. 2023). The great benefit of the direct measurement of energy stores before and after winter like in the present investigation is that the relation of different energy resources can be quantified, and that spring reserves can be determined. It has to be kept in mind, however, that measurements in autumn and spring cannot be done in the same individuals. Though such measurements have to be treated as snapshots of the selected populations, they provide reasonable estimations of winter energy use.

### The energy stores

Our quantification of the main energy stores in paper wasps overwintering in their natural hibernacula shows that they rely on fat as the main energy source (Fig. 1). This is similar to *Polistes metricus* where abdominal lipid stores were found to be considerably higher in autumn gynes than in foundress queens in spring, which in turn had higher stores than workers (summer individuals) (Toth et al. 2009). Judd (2018) also measured much higher abdominal fat stores in gynes than in workers in that species. Overwintering solitary *Osmia lignaria* bees also deplete their lipid stores during winter (Sgolastra et al. 2011), similar to *Culex pipiens*, the common house mosquito (Rozsypal et al. 2021).

Our investigation, however, shows that besides fat also glycogen is used to a considerable amount as winter energy fuel, though to a lower amount than lipids (Fig. 1). This resembles measurements in *Polistes metricus* (Judd 2018). One might assume glycogen to provide a sufficient supply of sugars, allowing the wasps to keep the level of free carbohydrates constant for direct metabolic use, because sugars from glycogen breakdown will enter the free carbohydrate pool. The measured ‘decrease’ of the free carbohydrate pool during overwintering (Fig. 1A4-C4; see also Tables 1 and 2) suggests that carbohydrate depletion is a more critical issue than depletion of lipid stores (Fig. 1A2-C2), because the rather low spring glycogen levels indicate that the wasps were nearly running out of their glycogen stores, especially *P. gallicus* IT (Fig. 1A3-C3).

### Effect of season and population on energy stores

The energetic challenge to get through the cold season is expected to differ between species from different climates (e.g., Kovac et al. 2022b). While in temperate climate it is the long duration of the winter season, the higher winter temperatures in Mediterranean climate may lead to premature depletion of energy stores. It is challenging to disentangle the different effects acting on the energy stores of different species in differing climates. Differences may result from different (genetic) adaptation to different climates, from (differential) acclimation to, or from direct physiological effects of current local climatic conditions. ANOVA showed that both population and season had a significant effect on mass-specific winter energy use (Table 4, Table S2). However, mass-specific effects of season and population on lipid and glycogen stores were mostly not completely independent from each other (Table S2). In addition, when comparing differences of mass-specific energy content between seasons, be it in relation to fresh mass or dry mass, there remains the problem that mass includes changing amounts of the energy resources under investigation between autumn and spring. Therefore, we calculated the content and seasonal change also in relation to ‘structure mass’ (SM), i.e., the difference between dry mass and measured mass of energy stores, as a somewhat more stable reference for comparison (Fig. 2). Again, ANOVA confirmed a significant effect of both population and season on lipid and glycogen energy content (Table S2). The lack of a population effect on the content of free carbohydrates (Table S2) indicates that this is a basic characteristic of paper wasp metabolism for proper supply of tissues with sugars, though the wasps obviously cannot prevent some decrease during winter hibernation.

We had expected the effect of season on energy content (Table 4, Table S2), with the highest absolute energy use in *P. dominula* AT but the highest mass-specific use in *P. dominula* IT (see Table 3 and Δ*J* in Fig. 2). The effect of population, however, turned out to be based on differences between *Polistes gallicus* IT and the two *P. dominula* populations only (Table 4). It was a surprising finding that the *P. dominula* populations overwintering in temperate Central European (Austrian) and warm Mediterranean (Italian) climate were not statistically different in their winter energy use, even if comparisons were done in relation to ‘structure mass’ (SM) (Table 4), though mean winter temperatures during the periods of investigation were ~ 5 °C higher in Italy: ~ 8.6 °C and ~ 3.6 °C in Italy and Austria as calculated from daily means of nearest weather stations. This lack of a statistical difference between the Austrian and Italian *P. dominula* coincides with calculations of winter energy use from respiratory curves and temperature measurements in hibernacula during a whole winter (Kovac et al. 2023). The lower respiratory metabolism (and sensitivity to temperature changes) of overwintering *P. dominula* IT gynes in comparison to *P. dominula* AT compensates for part of the higher metabolism caused by the higher winter temperatures experienced by *P. dominula* IT.

### Water content

Besides possible depletion of energy stores, the loss of water might be critical for the gynes’ survival of hibernation. However, a difference of water content between the autumn and spring samples of wasps was only observed in *P. dominula* AT but not in *P. dominula* IT and *P. gallicus* IT (Fig. 1, Table S7; see values in Table 1 and Table S1). In this context, it is of benefit for the wasps that they preferentially metabolize lipids in winter (3.3, 5.0, and 5.2 times the mass of total carbohydrates in the three populations, respectively; compare Tables 1 and 2). Oxidative metabolism of lipids provides nearly twice the amount of metabolic water (1.07 g water g^−1^ lipid) than carbohydrates (0.6 g water g^−1^ glucose) or proteins (0.5 g water g^−1^ protein; excretion of uric acid) (Dettner and Peters 2003). In a winter season, this amounts to about 12.5, 11.2, and 9.4 mg (µl) of metabolic water production from lipids and carbohydrates in *P. dominula* AT, *P. dominula* IT, and *P. gallicus* IT, and roughly 16, 15, and 14 mg (µl) if one adds water from proteins estimated according to the change of ‘structure mass’ (see below). Desiccation stress in late winter is therefore not likely to play a major role for survival. Due to the natural overwintering, our wasps had free access to the outside. Water uptake on warm days in late winter or early spring can therefore not be excluded. *Polistes biglumis* and *P. dominula* gynes were observed to leave their hibernacula for a while but return to them on a warm and sunny day in early spring (our own observation).

### Protein as energy resource in diapause

Beside lipids and carbohydrates, protein degradation has to be considered to contribute to energy supply during overwintering (Burmester 1999; Sinclair 2015). Sinclair and Marshall (2018) write that “In general, starved insects seem to switch from carbohydrate- to lipid- (and protein-)fuelled metabolism during the early stages of starvation …”. Measurements on this topic, however, are less abundant and precise than for lipids and carbohydrates. A diapause storage protein decline was reported in larvae of the southwestern corn borer *Diatraea grandiosella* (Chippendale 1973), and in adults of the Colorado potato beetle *Leptinotarsa decemlineata* (Lefevere et al. 1989) (see Hahn and Denlinger 2007). In a recent study, Shi et al. (2023) found a decrease of protein content of 10%, 2%, or 22% in the fat body of *Bombus terrestris* queens diapausing at 4 °C for 3 months, after 6 days of prediapause temperature acclimation at 10, 15, and 25 °C, respectively.

We noticed a lower ‘structure mass’ (i.e., dry mass minus total lipids and carbohydrates) in early spring than in autumn in those paper wasps sampled in the same winter season (*P. dominula* AT and *P. gallicus* IT, Table 1). If we suggest this to be mainly the result of protein degradation, this would contribute to energy supply to some extent. Calculation of a mean winter respiratory quotient (RQ_Winter_) as a weighted mean of measured lipid (L), glycogen (G), and free carbohydrate (C) changes between seasons (in mg; Table 1), results in a RQ_Winter_(L + G + C) = 0.77, 0.75 and 0.75 for *Polistes dominula* AT, *P. dominula* IT, and *P. gallicus* IT, respectively. If we add a (rough) estimate of protein (P) degradation from the ‘structure mass’ (SM) change of 3, (estimated) 3 and 4 mg, we get RQ_Winter_(L + G + C + P) = 0.78, 0.76 and 0.77, respectively. This is closer to the respiratory measurement of RQ_Winter_(resp.) = 0.78, 0.80 and 0.78 by Kovac et al. (2022b). The calculated mean daily winter energy use then even better resembles the range of model values calculated from respiratory curves and temperature recordings in winter hibernacula (Kovac et al. 2023) (see Fig. 3).

### Duration of life with spring reserves

An important question is how long paper wasps can keep up life with the resources remaining in spring. Sufficient energy is needed for basic subsistence, and for foraging flights for their own provisioning with food and for nest building. Kovac et al. (2022a) calculated that for resting metabolism the average energy use of a paper wasp during a whole summer season is about 1161 J g^−1^ fresh mass in *P. dominula* AT and 1522 J g^−1^ in *P.* gallicus IT, which equals to 104.5 J and 89.3 J per wasp for a mean spring mass of 90.0 mg and 58.7 mg, respectively. For a mixture of rest and nest activities, the calculation amounted to 121.5 J per wasp in *P. dominula* AT and 99.9 J per wasp in *P.* gallicus IT (1350.0 J g^−1^ and 1702.5 J g^−1^). The measured spring reserves (lipids and carbohydrates) of 396 J in *P. dominula* AT, 400 J in *P. dominula* IT, and 147 J in *P. gallicus* IT (Table 3, Fig. 2) will therefore allow them to keep up basic subsistence and nest activities like brood care etc. for at least an entire summer season.

Important tasks after winter dormancy, however, are orientation flights and foraging flights for the collection of food and nest material, and nest building. In this context, the question arises, how long the wasps could fly with their spring reserves? If we take the median flight metabolism of 34.54 ml O_2_ g^−1^ h^−1^ of *P. dominula* measured by Weiner et al. (2012), and the mean spring fresh mass of 90.0, 107.3, and 58.7 mg of our wasps, one estimates a mean flight energy turnover of (roughly) 18.23 mW in *P. dominula* AT, 21.73 mW in *P. dominula* IT and 11.89 mW in *P. gallicus* IT. The spring energy reserves from lipids and carbohydrates, therefore, would enable the wasps to continuously fly for *roughly* 6.03, 5.11 and 3.44 h, respectively. At first sight, this seems rather short. On the other hand, it may well suffice to find appropriate food if one considers that these wasps fly out only if environmental conditions are optimal for foraging (e.g., air temperature > ~ 18–20 °C). If conditions are bad, they can wait for long periods. We often observed them to crawl through the grass, on trees, or on the ground when searching for prey, which saves energy.

### Fuelling of initial spring and summer activity

The calculation of summer energy expenditure was done with the measured respiratory quotient of RQ_Summer_ = 1 of *Polistes dominula* and *P. gallicus* summer individuals (in contrast to RQ_Winter_ = 0.78–0.8 in overwintering gynes; Kovac et al. 2022b). This shows that carbohydrates are by far the main energetic fuel of summer adults, which contrasts to overwintering gynes which predominantly use lipids, and only about 31%, 20%, and 19% carbohydrates of lipid mass in *P. dominula* AT, *P. dominula* IT, and *P. gallicus* IT, respectively (compare Table 1). A similar change from a low winter RQ ~ 0.65–0.85 to a high summer RQ ~ 1 was also reported in the solitary bee *Osmia lignaria* by Sgolastra et al. (2010). We cannot exclude, however, that in paper wasps this change of RQ between seasons is a gradual one, with partial fat metabolism in the first phase of spring activity (compare Sgolastra et al. 2010). The question is, where the summer wasps get the carbohydrates from? On the one hand, it is well known that the Polistine wasps under investigation gather nectar (Kovac et al. 2019) and plant saps (Kovac and Stabentheiner 2001). On the other hand, provisioning of wasp adults by sweet salivary excretions of larvae has been reported a long time ago. Wheeler (1918) quoted a report by Roubaud (1916) that adults of *Belanogaster*, *Icaria* and *Polistes* “… are extremely eager for this salivary secretion, the taste of which is slightly sugary.”. This provisioning by (sweet) larval secretions was also reported in other wasps by Maschwitz (1966) and Brian and Brian (1952). It remains to be investigated, however, how the energy supply from fat reserves, which are still present in early spring (Fig. 1), works in detail in Polistine wasps (comp. Arrese and Soulages 2010), and whether they can use proline as a booster for flight carbohydrate metabolism like other hymenopterans (Teulier et al. 2016).

### Effect of climate change

If it comes to estimate the vulnerability of species to climate warming, the energy consumption is essential. Higher environmental temperatures may lead to later occurrence of low winter temperatures. Insects then will enter the energy-saving diapause later. Sgolastra et al. (2011) reported that overwintering solitary *Osmia lignaria* bees not only have lower pre-hibernation fat reserves but also lower post-diapause reserves the later they experience low winter temperatures. Kovac et al. (2023) reported that the *Polistes* winter hibernacula protect the wasps from predators, rain and snow but barely from environmental temperature. For a 2 °C increase of winter standard and hibernaculum temperature, they calculated additional costs of 33%, 30%, and 26% for *P. dominula* AT, *P. dominula* IT, and *P. gallicus* IT, respectively. For an extreme scenario of 3 °C temperature increase, an increase of winter metabolic expenditure of about 45%, 41%, and 36% has to be expected (Kovac et al. 2023). In view of the already approached global temperature increase on land of ~ 1.5 °C, this seems realistic. While overwintering gynes might be able to overcome these scenarios partially using more of their lipid reserves still present in spring, the depletion of their already low spring carbohydrate reserves might be the greater problem (Fig. 1, Table 2). For spring activity, the reduction of spring reserves will reduce the time window for first foraging flights. Though paper wasps were reported to flexibly adapt thermal brood care behavior to changing conditions (Stabentheiner et al. 2022), it remains unclear to what extent they are able to adapt their behavior and physiology to cope with unfavorable (warmer) future environment conditions, except by dispersing to cooler regions.

### Supplementary Information

Below is the link to the electronic supplementary material.

Supplementary file1 (PDF 3606 kb)

## Data Availability

Data are available in the manuscript or in the supplementary information.
