# Supplementary material for: The costs of overwintering in paper wasps (Polistes dominula and Polistes gallicus): the use of energy stores
Source: J Comp Physiol B. 2024 Mar 5;194(2):131–44. doi: 10.1007/s00360-024-01540-w (PMC11070328; doi:10.1007/s00360-024-01540-w)
Supplement: Supplementary file 1 — Supplementary file1 (PDF 3606 kb) [file 360_2024_1540_MOESM1_ESM.pdf]

The costs of overwintering in paper wasps (*Polistes dominula* and *Polistes gallicus*): the use of energy stores

Anton Stabentheiner[1 ‡], Teresa Mauerhofer[1], Regina Willfurth[1], Helmut Kovac[1 ‡], Edith Stabentheiner[1], Helmut Käfer[1], Iacopo Petrocelli[2]

[1]Institute of Biology, University of Graz, Universitätsplatz 2, 8010 Graz, Austria
[2]Dipartimento di Biologia, Università di Firenze, Via Madonna del Piano, 6 – 50019 Sesto Fiorentino, Italy
[‡]Authors for correspondence (anton.stabentheiner@uni-graz.at, helmut.kovac@uni-graz.at)

SUPPLEMENTARY MATERIALS

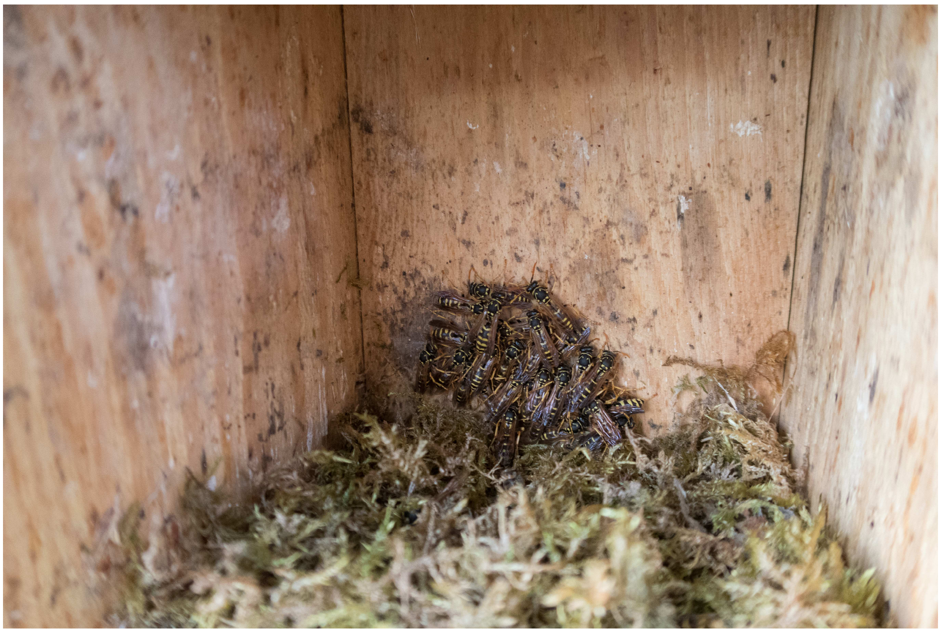

Fig. S1. Overwintering hibernaculum of *Polistes dominula*.

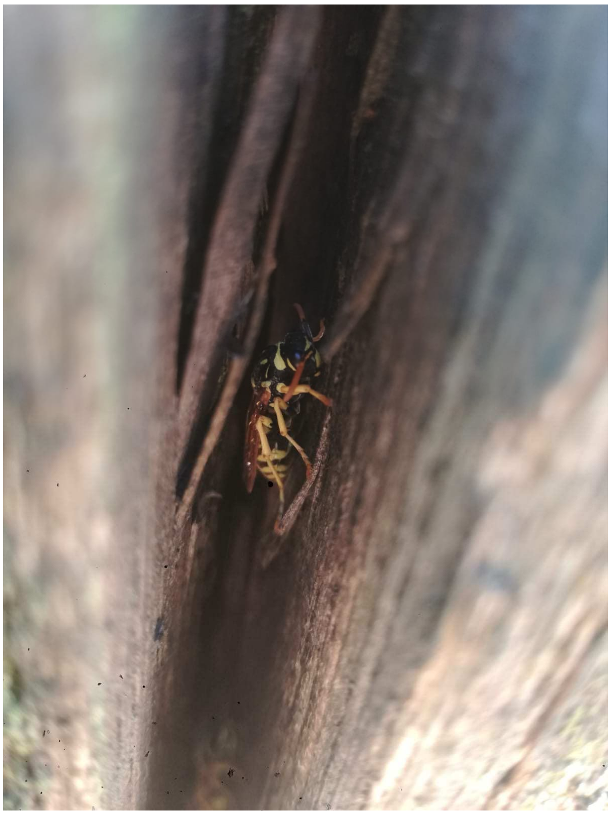

Fig. S2. Overwintering hibernaculum of *Polistes gallicus*.

Table S1. Extended descriptive statistics of energy resource determination in paper wasps *Polistes dominula* AT, *P. dominula* IT and *P. gallicus* IT (N = 148 wasps). Q1, Q3 = quartiles 1 and 3; Min, Max = minimum and maximum values.

Species/Population	*Season*	*Content per wasp (mg or %)*	*Min*	*Q1*	*Median*	*Q3*	*Max*	*Mean*	*SD*	*N*
***P. dominula* AT**	Autumn	Fresh mass (FM) (mg)	84.3	96.7	118.1	129.9	142.5	**113.1**	17.6	25
		Dry mass (DM) (mg)	35.1	43.4	50.0	59.0	68.7	**51.0**	9.1	25
		Water content (FM-DM) (mg)	45.4	53.8	65.3	70.4	76.5	**62.1**	9.7	25
		Structure mass (SM) (mg)	14.30	25.75	27.70	35.70	48.40	**30.35**	8.13	25
		Lipids (mg)	8.20	12.70	17.70	22.10	33.30	**17.86**	5.90	25
		Lipids (% of FM)	6.40	12.85	15.20	19.65	26.90	**16.01**	5.18	25
		Lipids (% of DM)	15.40	28.85	36.00	41.55	58.50	**35.32**	10.41	25
		Glycogen (mg)	0.93	1.65	2.24	3.38	5.08	**2.63**	1.31	24
		Glycogen (% of FM)	0.74	1.66	2.23	2.78	4.21	**2.28**	0.95	24
		Glycogen (% of DM)	1.53	3.82	4.95	6.19	9.97	**5.11**	2.20	24
		Free carbohydrates (mg)	0.07	0.17	0.22	0.30	0.60	**0.25**	0.13	25
		Free carbohydrates (% of FM)	0.07	0.16	0.22	0.24	0.50	**0.22**	0.10	25
		Free carbohydrates (% of DM)	0.14	0.34	0.48	0.55	1.15	**0.50**	0.22	25
***P. dominula* AT**	Spring	Fresh mass (FM) (mg)	49.1	78.9	87.9	98.0	135.4	**90.0**	17.3	45
		Dry mass (DM) (mg)	18.5	34.1	36.8	40.7	57.8	**37.9**	7.2	45
		Water content (FM-DM) (mg)	30.6	45.7	50.2	57.8	80.4	**52.1**	10.9	45
		Structure mass (SM) (mg)	14.30	23.75	27.10	30.95	46.30	**27.35**	6.43	45
		Lipids (mg)	2.70	6.70	9.40	13.25	21.80	**10.05**	4.28	45
		Lipids (% of FM)	2.80	7.90	10.70	13.55	25.20	**11.21**	4.48	45
		Lipids (% of DM)	7.20	18.65	26.00	31.55	58.60	**26.51**	10.30	45
		Glycogen (mg)	0.01	0.06	0.23	0.49	1.73	**0.37**	0.42	43
		Glycogen (% of FM)	0.02	0.08	0.27	0.46	1.47	**0.37**	0.38	43
		Glycogen (% of DM)	0.04	0.19	0.65	1.21	3.42	**0.89**	0.89	43
		Free carbohydrates (mg)	0.02	0.04	0.07	0.17	0.61	**0.13**	0.13	44
		Free carbohydrates (% of FM)	0.02	0.05	0.09	0.18	0.61	**0.14**	0.13	44
		Free carbohydrates (% of DM)	0.05	0.12	0.20	0.43	1.52	**0.33**	0.32	44
***P. dominula* IT**	Autumn	Fresh mass (FM) (mg)	84.3	85.1	87.1	109.8	118.3	**95.2**	13.2	10
		Dry mass (DM) (mg)	37.5	38.6	41.0	48.4	54.8	**43.2**	6.0	10
		Water content (FM-DM) (mg)	44.4	46.0	47.6	61.1	63.5	**52.0**	7.6	10
		Structure mass (SM) (mg)	19.20	19.83	21.75	27.68	52.50	**25.49**	10.09	10
		Lipids (mg)	15.00	16.25	17.50	19.25	20.00	**17.51**	1.70	9
		Lipids (% of FM)	16.10	17.25	18.00	21.45	23.20	**19.07**	2.47	9
		Lipids (% of DM)	36.90	38.65	40.00	46.20	48.40	**42.02**	4.14	9
		Glycogen (mg)	0.71	1.35	1.74	2.13	2.53	**1.68**	0.56	10
		Glycogen (% of FM)	0.83	1.45	1.80	2.18	2.32	**1.75**	0.49	10
		Glycogen (% of DM)	1.72	3.16	4.01	4.73	5.33	**3.88**	1.17	10
		Free carbohydrates (mg)	0.10	0.17	0.21	0.28	0.63	**0.25**	0.15	10
		Free carbohydrates (% of FM)	0.12	0.19	0.21	0.32	0.56	**0.26**	0.13	10
		Free carbohydrates (% of DM)	0.25	0.42	0.48	0.69	1.22	**0.57**	0.27	10
***P. dominula* IT**	Spring	Fresh mass (FM) (mg)	83.6	93.5	107.3	122.7	132.3	**107.3**	15.6	17
		Dry mass (DM) (mg)	31.8	35.7	39.7	45.8	48.0	**40.5**	5.4	17
		Water content (FM-DM) (mg)	51.3	56.5	65.4	76.3	86.7	**66.8**	11.2	17
		Structure mass (SM) (mg)	21.70	25.90	29.60	35.20	36.80	**29.97**	4.82	17
		Lipids (mg)	7.10	7.60	8.90	12.25	15.80	**10.10**	2.69	17
		Lipids (% of FM)	6.60	7.55	8.50	11.95	16.90	**9.56**	2.89	17
		Lipids (% of DM)	18.90	20.30	22.80	30.00	41.10	**24.95**	5.99	17
		Glycogen (mg)	0.20	0.20	0.30	0.50	0.90	**0.40**	0.26	17
		Glycogen (% of FM)	0.17	0.20	0.29	0.56	0.96	**0.38**	0.24	17
		Glycogen (% of DM)	0.43	0.55	0.78	1.40	2.33	**0.99**	0.58	17
		Free carbohydrates (mg)	0.02	0.03	0.04	0.07	0.11	**0.05**	0.03	17
		Free carbohydrates (% of FM)	0.01	0.03	0.04	0.07	0.12	**0.05**	0.03	17
		Free carbohydrates (% of DM)	0.04	0.08	0.10	0.17	0.29	**0.13**	0.08	17
***P. gallicus* IT**	Autumn	Fresh mass (FM) (mg)	51.9	63.2	66.0	77.1	99.5	**69.3**	11.3	25
		Dry mass (DM) (mg)	23.8	28.3	29.6	36.7	46.7	**32.1**	6.3	25
		Water content (FM-DM) (mg)	28.1	34.3	36.3	39.8	52.8	**37.2**	5.2	25
		Structure mass (SM) (mg)	14.90	18.35	20.40	24.65	28.50	**21.48**	3.93	25
		Lipids (mg)	4.30	7.25	8.80	10.75	18.00	**9.44**	3.16	25
		Lipids (% of FM)	6.70	11.65	13.80	16.00	18.10	**13.47**	3.07	25
		Lipids (% of DM)	14.70	26.15	28.90	34.55	38.60	**29.13**	6.24	25
		Glycogen (mg)	0.24	0.75	0.94	1.30	1.93	**1.04**	0.46	24
		Glycogen (% of FM)	0.37	1.14	1.49	1.86	2.50	**1.46**	0.56	24
		Glycogen (% of DM)	0.85	2.46	3.21	3.91	5.14	**3.14**	1.16	24
		Free carbohydrates (mg)	0.05	0.10	0.17	0.28	0.51	**0.20**	0.13	23
		Free carbohydrates (% of FM)	0.08	0.15	0.24	0.33	0.66	**0.27**	0.15	23
		Free carbohydrates (% of DM)	0.18	0.34	0.52	0.72	1.33	**0.57**	0.31	23
***P. gallicus* IT**	Spring	Fresh mass (FM) (mg)	37.1	52.7	57.8	65.4	85.5	**58.7**	9.3	26
		Dry mass (DM) (mg)	12.6	18.8	21.2	23.3	30.0	**21.1**	3.5	26
		Water content (FM-DM) (mg)	24.5	34.1	37.5	42.1	55.5	**37.7**	6.1	26
		Structure mass (SM) (mg)	10.60	15.63	18.00	19.45	25.20	**17.47**	3.00	26
		Lipids (mg)	1.80	2.88	3.15	4.33	6.40	**3.49**	1.08	26
		Lipids (% of FM)	3.00	5.08	5.45	6.35	11.40	**5.95**	1.59	26
		Lipids (% of DM)	8.60	14.20	15.70	17.58	33.10	**16.58**	4.52	26
		Glycogen (mg)	0.00	0.00	0.00	0.10	0.20	**0.03**	0.06	21
		Glycogen (% of FM)	0.01	0.02	0.05	0.10	0.29	**0.07**	0.07	21
		Glycogen (% of DM)	0.02	0.05	0.12	0.30	0.80	**0.20**	0.21	21
		Free carbohydrates (mg)	0.02	0.03	0.04	0.07	0.48	**0.07**	0.09	25
		Free carbohydrates (% of FM)	0.03	0.06	0.07	0.11	0.70	**0.11**	0.13	25
		Free carbohydrates (% of DM)	0.08	0.16	0.21	0.30	1.91	**0.30**	0.35	25

Table S2. ANOVA of mass-specific energy content of different energy resources, in J/g of fresh mass (FM), dry mass (DM) and structure mass (SM). Population: *Polistes dominula* AT, *Polistes dominula* IT, *Polistes gallicus* IT; Season: autumn and spring. Only measurements included where all three energy resources (lipids, glycogen, free carbohydrates) could be measured in the same individual (N = 134 wasps). Contrasts of lipid and glycogen energy content between populations significant between *P. dominula* AT and *P. gallicus* IT, and *P. dominula* IT and *P. gallicus* IT (P < 0.05). Contrasts of free carbohydrate energy content not different between populations (P > 0.05).

Energy resource	Fixed factors	Square sum	df	Mean squares	F value	P	N
**Lipids**	**Energy content (J/g) of FM**						134
	Population	32489561.1	2	16244780.6	7.288	0	
	Season	178894475	1	178894475	80.259	0	
	Population * Season	16422191	2	8211095.52	3.684	0.027	
	**Energy content (J/g) of DM**						134
	Population	204452657	2	102226329	10.41	0	
	Season	638692304	1	638692304	65.041	0	
	Population * Season	58202057.7	2	29101028.9	2.963	0.054	
	**Energy content (J/g) of SM**						134
	Population	1360306264	2	680153132	11.708	0	
	Season	4070224460	1	4070224460	70.064	0	
	Population * Season	580115295	2	290057647	4.993	0.008	
**Glycogen**	**Energy content (J/g) of FM**						134
	Population	166389.237	2	83194.618	14.333	0	
	Season	1751213.92	1	1751213.92	301.703	0	
	Population * Season	81053.876	2	40526.938	6.982	0.001	
	**Energy content (J/g) of DM**						134
	Population	963249.008	2	481624.504	16.069	0	
	Season	8306782.69	1	8306782.69	277.142	0	
	Population * Season	475559.161	2	237779.581	7.933	0	
	**Energy content (J/g) of SM**						134
	Population	3913706.21	2	1956853.1	21.355	0	
	Season	27018086.3	1	27018086.3	294.851	0	
	Population * Season	2268841.76	2	1134420.88	12.38	0	
**Free carbohydrates**	**Energy content (J/g) of FM**						134
	Population	891.36	2	445.68	1.006	0.368	
	Season	13349.888	1	13349.888	30.124	0	
	Population * Season	1210.536	2	605.268	1.366	0.258	
	**Energy content (J/g) of DM**						134
	Population	4009.931	2	2004.966	0.867	0.422	
	Season	54518.917	1	54518.917	23.585	0	
	Population *	5881.948	2	2940.974	1.272	0.283	
	**Energy content (J/g) of SM**						134
	Population	545.432	2	272.716	0.052	0.949	
	Season	229662.607	1	229662.607	44.08	0	
	Population * Season	24852.619	2	12426.309	2.385	0.095	

Table S3. Collection dates of *Polistes* gynes for determination of energy stores. #In spring 2020 any public movement (and thus collection of wasps) was prohibited by a severe covid-19 pandemic lockdown in Italy; ##calculation of days was done by subtracting 365 days from the actual difference between dates.

		Autumn	Spring	Days	Mean of days
Polistes dominula AT	Winter 1	21.11.2018	25.03.2019	124	129,5
	Winter 2	18.11.2019	01.04.2020	135	
Polistes dominula IT	Winter 1	–	–	–	132##
	Winter 2	15.11.2019	26.03.2021#	132##	
Polistes gallicus IT	Winter 1	21.11.2018	08.04.2019	138	138
	Winter 2	15.11.2019	– #	–	
					Total mean 133,17

Table S4. Descriptive statistics of data used for calculation of energy consumption in paper wasps *Polistes dominula* AT, *P. dominula* IT and *P. gallicus* IT (N = 134 wasps). Only measurements included where all three energy resources (lipids, glycogen and free carbohydrates) could be measured in the same individual. Q1, Q3 = quartiles 1 and 3; Min, Max = minimum and maximum values.

Species/Population	*Season*	*Energy source*	*Min*	*Q1*	*Median*	*Q3*	*Max*	*Mean*	*SD*	*N*
***P. dominula* AT**	**Autumn**	Lipids (J)	319.50	475.70	667.56	872.82	1296.41	**688.86**	232.75	24
		Lipids (J/g) of FM	2501.92	4938.12	5881.10	7393.81	10457.02	**6119.13**	1984.51	24
		Lipids (J/g) of DM	5973.21	11097.20	13984.88	15775.11	22745.34	**13602.83**	4077.73	24
		Lipids (J/g) of SM	7808.33	16178.42	24085.72	30217.18	62865.35	**24901.13**	12501.69	24
		Glycogen (J)	14.60	25.87	35.13	53.01	79.69	**41.33**	20.55	24
		Glycogen (J/g) of FM	115.68	259.17	349.56	436.40	661.52	**357.53**	149.51	24
		Glycogen (J/g) of DM	239.96	600.02	776.82	971.08	1565.65	**801.98**	345.22	24
		Glycogen (J/g) of SM	342.45	968.94	1433.76	1855.12	2921.77	**1400.81**	637.53	24
		Free carbohydrates (J)	1.65	2.76	3.56	4.84	9.42	**4.10**	1.94	24
		Free carbohydrates (J/g) of FM	16.01	26.59	34.03	37.90	78.45	**35.86**	14.73	24
		Free carbohydrates (J/g) of DM	33.21	58.00	77.53	86.20	181.18	**80.21**	32.58	24
		Free carbohydrates (J/g) of SM	47.39	103.69	137.31	166.80	256.02	**138.12**	53.31	24
		Total (J)	399.82	518.22	711.71	915.34	1351.38	**734.30**	234.99	24
		Total (J/g) of FM	3145.93	5246.44	6413.05	7923.04	10843.01	**6512.52**	1973.55	24
		Total (J/g) of DM	7252.25	11615.92	14908.11	16900.83	23584.92	**14485.03**	4028.20	24
		Total (J/g) of SM	9480.32	17195.33	25421.19	32439.18	65185.85	**26440.06**	12783.12	24
***P. dominula* AT**	**Spring**	Lipids (J)	103.33	260.80	365.95	507.66	848.45	**387.84**	162.75	42
		Lipids (J/g) of FM	1075.28	3027.42	4129.58	5187.13	9797.39	**4254.38**	1628.17	42
		Lipids (J/g) of DM	2807.99	7071.33	10094.21	11692.97	22807.90	**10065.13**	3778.96	42
		Lipids (J/g) of SM	3080.72	8666.57	13785.26	17298.37	56562.24	**14961.24**	9230.99	42
		Glycogen (J)	0.21	0.92	3.44	7.62	27.21	**5.74**	6.61	42
		Glycogen (J/g) of FM	2.81	12.16	40.50	69.64	230.60	**56.83**	59.01	42
		Glycogen (J/g) of DM	6.24	28.11	97.79	187.45	536.70	**135.93**	138.96	42
		Glycogen (J/g) of SM	8.50	37.23	129.87	271.30	850.29	**194.78**	204.61	42
		Free carbohydrates (J)	0.27	0.63	1.15	2.80	9.53	**2.05**	2.16	42
		Free carbohydrates (J/g) of FM	3.37	8.16	12.64	26.92	95.38	**21.28**	21.25	42
		Free carbohydrates (J/g) of DM	7.92	19.28	29.85	68.94	239.41	**50.86**	51.16	42
		Free carbohydrates (J/g) of SM	9.71	24.59	40.79	99.77	329.90	**71.96**	72.49	42
		Total (J)	112.78	274.09	371.32	523.72	854.55	**395.62**	165.89	42
		Total (J/g) of FM	1173.53	3117.02	4206.31	5256.86	9867.82	**4332.48**	1632.25	42
		Total (J/g) of DM	3064.57	7176.88	10210.44	12061.63	22971.86	**10251.92**	3793.30	42
		Total (J/g) of SM	3362.22	8946.09	13927.81	17844.33	56968.85	**15227.98**	9286.71	42
***P. dominula* IT**	**Autumn**	Lipids (J)	584.22	630.83	680.94	748.82	777.79	**680.93**	66.41	9
		Lipids (J/g) of FM	6252.90	6713.14	7016.03	8339.51	9033.60	**7421.15**	960.30	9
		Lipids (J/g) of DM	14365.85	15028.03	15579.19	17964.64	18832.75	**16347.15**	1608.27	9
		Lipids (J/g) of SM	25095.79	26101.49	28447.63	36722.97	38342.23	**30879.71**	5269.72	9
		Glycogen (J)	11.18	18.84	26.45	31.43	39.67	**25.55**	8.88	9
		Glycogen (J/g) of FM	129.85	205.56	284.30	345.31	364.31	**273.74**	81.94	9
		Glycogen (J/g) of DM	270.70	447.22	645.17	770.19	837.00	**609.20**	194.25	9
		Glycogen (J/g) of SM	551.13	778.33	1169.42	1475.12	1513.84	**1142.10**	368.90	9
		Free carbohydrates (J)	1.54	2.73	3.50	4.55	9.87	**4.00**	2.40	9
		Free carbohydrates (J/g) of FM	18.25	30.60	33.79	52.27	87.74	**42.09**	20.38	9
		Free carbohydrates (J/g) of DM	38.56	70.11	77.64	110.83	191.66	**92.58**	43.44	9
		Free carbohydrates (J/g) of SM	80.17	129.25	136.20	209.90	335.94	**171.83**	75.92	9
		Total (J)	611.15	653.16	711.42	777.44	804.47	**710.48**	67.43	9
		Total (J/g) of FM	6651.01	7071.92	7227.02	8694.03	9219.55	**7736.99**	929.08	9
		Total (J/g) of DM	15280.49	15623.43	16389.73	18728.82	19220.42	**17048.94**	1542.67	9
		Total (J/g) of SM	26693.58	27137.89	30088.12	38021.30	39657.28	**32193.64**	5261.97	9
***P. dominula* IT**	**Spring**	Lipids (J)	276.29	295.29	347.99	477.00	615.08	**392.93**	104.78	17
		Lipids (J/g) of FM	2569.19	2934.96	3289.40	4650.23	6592.47	**3718.27**	1125.68	17
		Lipids (J/g) of DM	7345.92	7884.80	8850.71	11665.08	15976.03	**9700.82**	2333.63	17
		Lipids (J/g) of SM	9165.21	9987.52	11554.83	16848.79	28371.18	**13515.91**	4931.79	17
		Glycogen (J)	2.44	3.58	5.42	8.20	14.08	**6.37**	3.94	17
		Glycogen (J/g) of FM	27.18	30.77	44.81	87.33	150.89	**60.10**	38.24	17
		Glycogen (J/g) of DM	67.48	86.06	122.43	218.84	365.67	**155.19**	91.02	17
		Glycogen (J/g) of SM	97.28	113.90	156.97	302.29	649.37	**218.62**	152.28	17
		Free carbohydrates (J)	0.26	0.42	0.58	0.99	1.76	**0.78**	0.48	17
		Free carbohydrates (J/g) of FM	2.30	4.11	6.20	10.31	18.83	**7.73**	5.35	17
		Free carbohydrates (J/g) of DM	6.60	11.97	16.45	26.30	45.63	**19.78**	12.63	17
		Free carbohydrates (J/g) of SM	8.20	15.54	21.12	35.65	81.04	**28.04**	20.65	17
		Total (J)	280.50	302.51	352.23	483.10	630.91	**400.08**	107.45	17
		Total (J/g) of FM	2598.76	2978.62	3355.28	4698.76	6762.19	**3786.10**	1153.45	17
		Total (J/g) of DM	7462.43	8001.90	8952.76	11787.37	16387.33	**9875.80**	2390.03	17
		Total (J/g) of SM	9270.81	10141.54	11688.06	17025.44	29101.59	**13762.56**	5056.32	17
***P. gallicus* IT**	**Autumn**	Lipids (J)	168.56	283.49	344.07	430.83	701.23	**373.97**	125.64	22
		Lipids (J/g) of FM	2609.37	4525.31	5198.59	6146.63	7047.54	**5231.10**	1122.72	22
		Lipids (J/g) of DM	5714.07	10256.87	11119.51	13255.74	15015.64	**11295.62**	2238.77	22
		Lipids (J/g) of SM	7103.06	14240.77	16471.18	22245.81	25940.35	**17244.11**	4776.75	22
		Glycogen (J)	3.75	11.87	16.18	21.20	30.33	**16.87**	7.14	22
		Glycogen (J/g) of FM	58.72	179.27	247.11	301.48	391.81	**236.51**	86.49	22
		Glycogen (J/g) of DM	134.01	388.07	529.13	629.28	807.39	**509.80**	179.85	22

Species/Population	Season	Energy source	Min	Q1	Median	Q3	Max	Mean	SD	N
		Glycogen (J/g) of SM	185.72	517.82	819.32	929.72	1339.54	**775.98**	307.28	22
		Free carbohydrates (J)	0.77	1.48	2.60	4.56	8.04	**3.13**	2.05	22
		Free carbohydrates (J/g) of FM	13.07	22.84	38.64	53.19	103.81	**42.53**	24.31	22
		Free carbohydrates (J/g) of DM	27.59	51.38	83.38	115.23	208.71	**90.85**	49.42	22
		Free carbohydrates (J/g) of SM	42.93	75.81	112.55	182.08	350.42	**139.14**	84.11	22
		Total (J)	191.10	295.76	361.51	458.43	727.00	**393.97**	131.17	22
		Total (J/g) of FM	2958.22	4642.90	5516.66	6506.60	7306.49	**5510.15**	1163.55	22
		Total (J/g) of DM	6477.99	10509.77	11737.49	14234.09	15567.36	**11896.27**	2302.83	22
		Total (J/g) of SM	8052.68	14592.37	17410.89	23459.68	26893.48	**18159.23**	4984.30	22
***P. gallicus* IT**	**Spring**	Lipids (J)	71.31	113.32	139.84	176.49	248.16	**145.15**	42.23	20
		Lipids (J/g) of FM	1169.01	1964.48	2157.58	2690.52	4439.31	**2371.87**	694.44	20
		Lipids (J/g) of DM	3347.87	5498.72	6118.59	7110.22	12857.89	**6562.45**	1974.54	20
		Lipids (J/g) of SM	3693.54	6420.26	7298.24	8754.32	19376.47	**8133.82**	3256.58	20
		Glycogen (J)	0.07	0.17	0.33	1.19	3.18	**0.71**	0.79	20
		Glycogen (J/g) of FM	1.08	2.78	5.98	16.23	46.23	**11.55**	12.11	20
		Glycogen (J/g) of DM	3.05	7.67	17.02	46.73	125.72	**31.83**	33.02	20
		Glycogen (J/g) of SM	3.95	8.95	21.36	66.71	156.58	**38.61**	40.03	20
		Free carbohydrates (J)	0.27	0.52	0.77	1.23	7.60	**1.18**	1.56	20
		Free carbohydrates (J/g) of FM	5.16	9.74	12.78	18.66	110.47	**18.32**	22.36	20
		Free carbohydrates (J/g) of DM	12.36	25.63	35.48	53.21	300.41	**50.82**	60.84	20
		Free carbohydrates (J/g) of SM	14.86	29.99	45.13	67.70	374.15	**62.49**	76.06	20
		Total (J)	73.83	114.15	140.91	179.35	249.94	**147.04**	42.58	20
		Total (J/g) of FM	1210.27	1989.88	2188.07	2704.36	4471.18	**2401.74**	694.88	20
		Total (J/g) of DM	3466.02	5531.48	6186.72	7218.36	12950.21	**6645.10**	1976.11	20
		Total (J/g) of SM	3823.90	6458.52	7379.55	8929.41	19515.61	**8234.93**	3266.82	20

Table S5. Post-hoc tests of a) effect of season and b) differences between species or populations of fresh mass (FM), dry mass (DM), water content (FM-DM), and percent content of lipids, glycogen and free (soluble) carbohydrates. * P < 0.05; ** P < 0.01; *** P < 0.001 (Mann-Whitney U test).

a) Seasonal changes	***Parameter***	***P***	
***P. dominula* AT Autumn** (N=25) **– *P. dominula* AT Spring** (N=45)	FM [mg]	0.000	***
	DM [mg]	0.000	***
	FM-DM [mg]	0.000	***
	Lipids (% of FM)	0.000	***
	Lipids (% of DM)	0.000	***
	Glycogen (% of FM)	0.000	***
	Glycogen (% of DM)	0.000	***
	Free carbohydrates (% of FM)	0.000	***
	Free carbohydrates (% of DM)	0.000	***
***P. dominula* IT Autumn** (N=10) **– *P. dominula* IT Spring** (N=17)	FM [mg]	0.052	
	DM [mg]	0.334	
	FM-DM [mg]	0.001	**
	Lipids (% of FM)	0.000	***
	Lipids (% of DM)	0.000	***
	Glycogen (% of FM)	0.000	***
	Glycogen (% of DM)	0.000	***
	Free carbohydrates (% of FM)	0.000	***
	Free carbohydrates (% of DM)	0.000	***
***P. gallicus* IT Autumn** *(N=25)* **– *P. gallicus* IT Spring** (N=26)	FM [mg]	0.001	**
	DM [mg]	0.000	***
	FM-DM [mg]	0.658	
	Lipids (% of FM)	0.000	***
	Lipids (% of DM)	0.000	***
	Glycogen (% of FM)	0.000	***
	Glycogen (% of DM)	0.000	***
	Free carbohydrates (% of FM)	0.000	***
	Free carbohydrates (% of DM)	0.000	***
b) Differences between populations or species	***Parameter***	***P***	
***P. dominula* AT Autumn** (N=25) **– *P. dominula* IT Autumn** (N=10)	FM [mg]	0.007	**
	DM [mg]	0.019	*
	FM-DM [mg]	0.007	**
	Lipids (% of FM)	0.060	
	Lipids (% of DM)	0.027	*
	Glycogen (% of FM)	0.109	
	Glycogen (% of DM)	0.101	
	Free carbohydrates (% of FM)	0.627	
	Free carbohydrates (% of DM)	0.653	
***P. dominula* AT Autumn** (N=25) **– *P. gallicus* IT Autumn** (N=25)	FM [mg]	0.000	***
	DM [mg]	0.000	***
	FM-DM [mg]	0.000	***
	Lipids (% of FM)	0.056	
	Lipids (% of DM)	0.007	**

	Glycogen (% of FM)	0.001	**
	Glycogen (% of DM)	0.001	**
	Free carbohydrates (% of FM)	0.420	
	Free carbohydrates (% of DM)	0.529	
***P. dominula* IT Autumn** (N=10) **– *P. gallicus* IT Autumn** (N=25)	FM [mg]	0.000	***
	DM [mg]	0.000	***
	FM-DM [mg]	0.000	***
	Lipids (% of FM)	0.000	***
	Lipids (% of DM)	0.000	***
	Glycogen (% of FM)	0.148	
	Glycogen (% of DM)	0.079	
	Free carbohydrates (% of FM)	0.463	
	Free carbohydrates (% of DM)	0.731	
***P. dominula* AT Spring** (N=45) **– *P. dominula* IT Spring** (N=17)	FM [mg]	0.001	**
	DM [mg]	0.115	
	FM-DM [mg]	0.000	***
	Lipids (% of FM)	0.136	
	Lipids (% of DM)	0.619	
	Glycogen (% of FM)	0.371	
	Glycogen (% of DM)	0.168	
	Free carbohydrates (% of FM)	0.000	***
	Free carbohydrates (% of DM)	0.001	**
***P. dominula* AT Spring** (N=45) **– *P. gallicus* IT Spring** (N=26)	FM [mg]	0.000	***
	DM [mg]	0.000	***
	FM-DM [mg]	0.000	***
	Lipids (% of FM)	0.000	***
	Lipids (% of DM)	0.000	***
	Glycogen (% of FM)	0.000	***
	Glycogen (% of DM)	0.000	***
	Free carbohydrates (% of FM)	0.463	
	Free carbohydrates (% of DM)	0.731	
***P. dominula* IT Spring** (N=17) **– *P. gallicus* IT Spring** (N=26)	FM [mg]	0.000	***
	DM [mg]	0.000	***
	FM-DM [mg]	0.000	***
	Lipids (% of FM)	0.000	***
	Lipids (% of DM)	0.000	***
	Glycogen (% of FM)	0.000	***
	Glycogen (% of DM)	0.000	***
	Free carbohydrates (% of FM)	0.001	**
	Free carbohydrates (% of DM)	0.000	***

DETAILED EXTRACTION PROCEDURES of Lipids, Glycogen and Free Carbohydrates.

The determination of energy resources (lipids, glycogen and free carbohydrates) followed the protocol of Jana C. Lee (2019), with some adaptations according to Lorenz (2003) and Guckert and White (1988).

All steps of sample preparation, which require the use of organic solvents, have to be done with appropriate safety precaution! This refers to the safe storage and disposal of chemicals, working under laboratory hoods, and use of appropriate protective clothing (working with hot acids required!).

A) Sample preparation:

The frozen wasps are equilibrated to room temperature for one hour in the Eppendorf vials. Afterwards their fresh mass (FM) is determined. After drying them at 55 °C for three days in an oven with recirculating air their dry mass (DM) is measured.

Before being used for analysis the wasps are swirled in n-hexane for 30 seconds to wash off cuticular lipids and this way improve determination of storage lipids, and dried for 20 minutes at room temperature.

Every now dried wasp is cut to small pieces with scissors in the Eppendorf vial, and then milled in the vial with three steel bullets (diameter 2 mm) in a Retsch mill for two minutes at 30 Hz (room temperature). The result is a fine-grained powder promising optimal extraction.

B) Extraction procedures:

Chemicals

Aqua bidest
Isopropanol (C_3H_8O)
Sodium sulphate (Na_2SO_4)
n-Hexane (C_6H_{14})

Solutions

Extraction reagent: *n*-Hexane:Isopropanol = 3:2
Saturated Na_2SO_4: 10g Na_2SO_4 in 50 ml Aqua bidest

For lipid and free carbohydrate extraction 600 µl of extraction reagent and 100 µl Na_2SO_4 are added to each vial, which afterwards is vortexed for 30 seconds, shaken for 5 minutes in a thermomixer, and then centrifuged for 15 min at 14,000 rpm (all at room temperature). The supernatant is pipetted in a new 2 ml Eppendorf vial. The remainder is extracted again with 400 µl reagent, the supernatant added to the first supernatant and filled up to 1 ml if necessary, and stored at 4 °C. **The combined supernatant is divided in two parts (1:1 for lipids and free carbohydrates)**, and dried by evaporation in a Speed Vac (temperature set to "low") for four hours.

The pellet (solid phase) in the original extraction vial is dried at 50 °C in a thermomixer, and **used for glycogen determination**.

All Eppendorf vials are purged with nitrogen (N_2) and stored at -80 °C for later use.

B1) Lipid extraction (Phosphovanillin reaction).

Chemicals

Lipid standard-Mix: 1 mg/ml Tripalmitin ($C_{51}H_{98}O_6$) 41%,
1 mg/ml Triolein ($C_{57}H_{104}O_6$) 36%,
1 mg/ml Trilinolenin 23% ($C_{57}H_{98}O_6$) in n-Hexane (C_6H_{14})
Phosphoric acid (H_3PO_4)
Sulphuric acid (H_2SO_4)
Vanillin ($C_8H_8O_3$)

Solutions

Phosphovanillin (PV) reagent:
Solve 70 mg Vanillin in 10 ml Aqua bidest, then (slowly) add 40 ml of 85% phosphoric acid (possibly on ice). *The reagent keeps quality at room temperature for months if stored in the dark (wrap in aluminum foil). Has to be replaced if colour changes to pink.*

Lipid content is determined with the Sulfophosphovanillin reaction according to Park et al. (2016). Its high reliability was proved by Williams et al. (2011). As a calibration standard a mix of 1 mg ml^{-1} tripalmitin ($C_{51}H_{98}O_6$) 41%, 1 mg ml^{-1} triolein ($C_{57}H_{104}O_6$) 36%, and 1 mg ml^{-1} trilinolein 23% ($C_{57}H_{98}O_6$) in n-hexane (C_6H_{14}) can be used.

For lipid extraction a mixture of hexane and isopropanol (3:2) is used, according to Guckert et al. (1988), Guckert and White (1988) and Palmquist and Jenkins (2003), instead of chloroform and methanol (Lee 2019; Lorenz 2003). Hexane and isopropanol extracts a smaller portion of polar lipids, and this way of phospholipids in cell membranes, in comparison to chloroform and methanol (Guckert et al. 1988; Guckert and White 1988; Palmquist and Jenkins 2003), and this way improves determination of storage lipids.
The **fine-grained reaction powder produced with the Retsch mill** promises an accurate extraction of storage lipids.

The **first part of the combined supernatant from the sample preparation** (the second half can be used for determination of free carbohydrates) is dissolved in 1 ml n-hexane and vortexed for 30 seconds. 200 µl of this solution are added to 800 µl n-hexane (1:4).

Another 30 µl of the solution are pipetted into a new Eppendorf vial and dried in a thermomixer for 5 minutes at 55 °C. After drying, 100 µl of concentrated sulfuric acid is added. Then the samples are incubated for 17 minutes at 90 °C in a thermomixer and eventually cooled on ice. After adding 1,900 µl PV reagent the samples are shaken and allowed to react for 15 minutes in the dark.

The lipid standard is thawed, vortexed shortly and pipetted into Eppendorf vials in concentrations of 0 µg ml^{-1}, 10 µg ml^{-1}, 20 µg ml^{-1}, 30 µg ml^{-1} and 40 µg ml^{-1}, and otherwise processed like the wasp samples.

The sample and the standard solutions are measured in disposable PMMA cuvettes in a photometer at 530 nm wavelength, with air as a reference.

The lipid sample parts not used for analysis are dried for four hours in a Speed Vac (temperature set to "low"), purged with nitrogen (N_2), and stored at -80 °C for possible future use.

Calculation:

$$\frac{1000\ \text{(solvent)} * \text{Concentration (photometer)}}{30\ \text{(sample quantity)}} * 5\ \text{(dilution)} * 2\ \text{(separated sample)} = \mathbf{\mu g\ Lipid\ per\ wasp}$$

B2) Glycogen (Anthrone reaction).

Chemicals

Anthrone ($C_{14}H_{10}O$)
Glycogen ($(C_6H_{10}O_5)_n$): Stock solution 1 mg ml^{-1} Glycogen in Aqua bidest (always prepare freshly)
Methanol 80% (CH_3OH)
Sulphuric acid (H_2SO_4)

Solutions

Anthrone reagent:

The Anthrone reagent is prepared by adding 380 ml concentrated H_2SO_4 slowly to 150 ml of Aqua bidest (water first!) in a stirred ice bath (attention: becomes quite hot!). Afterwards, 750 mg of Anthrone is added to the hot solution. *The Anthrone reagent should always be prepared in the amounts described here, because dissolving may remain incomplete in smaller amounts (probably because otherwise the sulphuric acid cools down too fast)! The yellow Anthrone reagent is stable for about 4 months if kept at 4 °C in the dark (no longer usable if the colour changes to bluish-green).*

Glycogen standard: Stock solution: 1 mg ml^{-1} (always prepare new).

Solve glycogen and heat to 70 °C for ca. 3 minutes.

For determination of the glycogen content the **pellet (solid phase) of the original extraction** is washed with 400 µl methanol, vortexed for 30 seconds and then centrifuged for 5 minutes at 10,000 rpm. The supernatant is removed. Then the procedure is repeated.

The resulting (washed) pellets are now incubated with 500 µl Aqua bidest at 90 °C for 4 minutes. To prevent bursting open of the lid the Eppendorf vials are allowed to cool somewhat down, and then are centrifuged at 3,000 rpm for 1 minute. The supernatant is pipetted into a new vial. The procedure is repeated once again and the supernatant joined with the first one. The reaction vial is then filled up to 1 ml with Aqua bidest.

For calibration the glycogen standard has to be prepared freshly, and pipetted into 2 ml Eppendorf vials in concentrations of 0 µg ml^{-1}, 25 µg ml^{-1}, 50 µg ml^{-1} and 100 µg ml^{-1}.

100 µl of the samples as well as of the different concentrations of the calibration standard are pipetted into graded glass vials and filled up to 5 ml with the Anthrone reagent. After incubating the glass vials for 15 minutes at 90 °C, they are cooled down to room temperature with cold water. The solution is transferred to disposable PMMA cuvettes and measured in a photometer at 620 nm, with air as a reference.

The glycogen standard has always to be prepared freshly, at a concentration of 1 mg glycogen ml^{-1}, by solving the glycogen in Aqua bidest and heating the solution to 70 °C for three minutes.

Calculation:

$$\frac{1000\ \text{(solvent)} * \text{Concentration (photometer)}}{100\ \text{(sample quantity)}} = \mathbf{\mu g\ Glycogen\ per\ wasp}$$

B3) Free (soluble) carbohydrates (Anthrone reaction).

Chemicals

Anthrone ($C_{14}H_{10}O$)
Glucose ($(C_6H_{12}O_6)_n$: Stock solution: 1 mg ml^{-1} in 25% ethanol
Sulphuric acid (H_2SO_4)

Solutions

Anthrone reagent:

The Anthrone reagent is prepared by adding 380 ml concentrated H_2SO_4 slowly to 150 ml of Aqua bidest (water first!) in a stirred ice bath (attention: becomes quite hot!). Afterwards, 750 mg of Anthrone is added to the hot solution. *The Anthrone reagent should always be prepared in the amounts described here, because dissolving may remain incomplete in smaller amounts (probably because otherwise the sulphuric acid cools down too fast)! The yellow Anthrone reagent is stable for about 4 months if kept at 4 °C in the dark (no longer usable if the colour changes to bluish-green).*

Glucose standard: Stock solution: 1 mg ml^{-1} in 25% ethanol (always prepare new).

500 µl of Aqua bidest is added to the **second part of the combined supernatant from the sample preparation** (the other half was used for lipid determination), vortexed for one minute, and the sample allowed to dissolve for 5 minutes in an ultrasonic bath. 100 µl of the resulting milky-turbid solution are pipetted into 2 ml reaction vials and added by 1,900 µl of Anthrone reagent.

The frozen glucose standard is thawed, vortexed shortly, pipetted into 2 ml Eppendorf vials in concentrations of 0 µg ml^{-1}, 10 µg ml^{-1}, 20 µg ml^{-1}, 30 µg ml^{-1} and 40 µg ml^{-1}, and each vial filled up to 2 ml with Anthrone reagent for generating a calibration curve in the photometer.

The sample and the standard solutions are incubated for 20 minutes at 90 °C in the thermomixer, and then cooled in cold water. The sample and standard solutions are measured in disposable PMMA cuvettes in a photometer at 620 nm, with air as a reference.

The rest of the free carbohydrate sample parts not used for analysis are dried for four hours by evaporation in a Speed Vac (temperature set to "low"), purged with nitrogen (N_2) and stored at -80 °C for possible future use.

Calculation:

$$\frac{500\ \text{(solvent)} * \text{Concentration (photometer)}}{100\ \text{(sample quantity)} * 2} = \textbf{µg Free (soluble) Carbohydrates per wasp}$$

C) Sources of chemicals:

Anthrone, Sigma 319899
D(+)-Glucose anhydrous (f. bioch. purpose), Merck 1.08337
Glycogen (for biochem. purpose), Merck 4202
n-Hexane p.a., Roth 7339.2
Sodium sulphate anhydrous >99%, Roth 8631.2
2-Propanol p.a., Roth 6752.2
o-Phosphoric acid 85% p.a., Merck 573
Sulphuric acid 95-97% z.A., Merck 1.00731
Trilinolenin (glyceryl trilinolenate), Sigma Aldrich T6513
Triolein (glyceryl trioleate), Sigma Aldrich T7140
Tripalmitin (glyceryl tripalmitate), Sigma Aldrich T5888
Vanillin DAB7, Merck 8510
